# Supplementary material for: Resilience of self-reported life satisfaction: A case study of who conforms to set-point theory in Australia
Source: PLoS One. 2020 Aug 13;15(8):e0237161. doi: 10.1371/journal.pone.0237161 (PMC7425895; doi:10.1371/journal.pone.0237161)
Supplement: S1 Table — This table summarizes the primary variables for our 3 subsamples and the whole sample. Columns 1–4 provide the average across individuals (LS¯i) of the average LS within an individual over time (LSit) [see Eq 1]. The (parenthesis) provide the standard deviation between the average LS across individuals. Columns 5–8 provide the average across individuals of the standard deviations within each individual over time (σiLS) [see Eq 2]. The (parenthesis) provide the standard deviation among the standard deviations of each individual over time. (PDF) [file pone.0237161.s001.pdf]

	Within person averages over time *(SD between averages of LS of individuals)*				Within person SDs over time *(SD between SD of individuals)*			
	**Suffering**	**Struggling**	**Thriving**	**All**	**Suffering**	**Struggling**	**Thriving**	**All**
	**(1)**	**(2)**	**(3)**	**(4)**	**(5)**	**(6)**	**(7)**	**(8)**
N	37	474	5,476	5,987	37	474	5,476	5,987
Life satisfaction	3.55	5.84	8.13	7.92	0.57	0.52	0.79	1.05
	(1.93)	(1.47)	(0.84)	(0.90)	(0.60)	(0.55)	(0.40)	(0.45)
Extroversion	3.85	4.05	4.41	4.38	1.11	0.90	0.98	0.99
	(0.59)	(0.49)	(0.47)	(0.47)	(0.34)	(0.27)	(0.26)	(0.26)
Agreeableness	5.25	5.19	5.47	5.45	1.03	0.85	0.76	0.77
	(0.62)	(0.53)	(0.47)	(0.48)	(0.38)	(0.35)	(0.31)	(0.32)
Conscientiousness	4.44	4.82	5.22	5.19	1.03	0.94	0.88	0.89
	(0.66)	(0.52)	(0.48)	(0.49)	(0.42)	(0.30)	(0.29)	(0.29)
Emotional stability	4.27	4.79	5.37	5.32	1.22	0.86	0.90	0.92
	(0.68)	(0.61)	(0.54)	(0.55)	(0.64)	(0.35)	(0.32)	(0.33)
Openness	4.29	4.33	4.17	4.18	1.25	1.02	0.95	0.96
	(0.52)	(0.52)	(0.50)	(0.50)	(0.26)	(0.34)	(0.29)	(0.29)
HH disposable income	44,856	61,857	82,102	80,269	23,492	34,005	54,502	53,378
	(31,502)	(31,871)	(39,193)	(38,565)	(40,362)	(30,377)	(47,278)	(46,167)
Age	50.05	47.80	50.55	50.33	12.20	12.10	14.73	14.54
	(5.05)	(5.05)	(5.05)	(5.05)	(0.00)	(0.00)	(0.00)	(0.00)

Table S1: Summary statistics, BALANCED sample. This table summarizes the primary variables for our 3 subsamples and the whole sample. Columns 1-4 provide the average across individuals ($\overline{LS}_i$) of the average LS within an individual over time (LS_{it}) [see equation 1]. The *(parenthesis)* provide the standard deviation between the average LS across individuals. Columns 5-8 provide the average across individuals of the standard deviations within each individual over time (σ_i^{LS}) [see equation 2]. The *(parenthesis)* provide the standard deviation among the standard deviations of each individual over time.
