# Supplementary material for: Resilience of self-reported life satisfaction: A case study of who conforms to set-point theory in Australia
Source: PLoS One. 2020 Aug 13;15(8):e0237161. doi: 10.1371/journal.pone.0237161 (PMC7425895; doi:10.1371/journal.pone.0237161)
Supplement: S2 Table — Comparison between results from main (unbalanced) sample and the strongly balanced sample. (PDF) [file pone.0237161.s002.pdf]

	(1) Unbalanced (8 – 17 waves)	(2) Balanced (all 17 waves)
Life satisfaction (mean)	-0.216***	-0.213***
	(0.00478)	(0.00614)
Extroversion (mean)	0.0223***	0.0244***
	(0.00394)	(0.00498)
Agreeableness (mean)	0.0501***	0.0692***
	(0.00579)	(0.00790)
Conscientious (mean)	0.00455	0.00871
	(0.00453)	(0.00628)
Emotional stability (mean)	-0.0157**	-0.0118
	(0.00497)	(0.00659)
Openness to experience (mean)	-0.00998*	-0.00446
	(0.00427)	(0.00565)
Extroversion (std.dev.)	0.119***	0.121***
	(0.0138)	(0.0208)
Agreeableness (std.dev.)	0.104***	0.137***
	(0.0139)	(0.0200)
Conscientious (std.dev.)	0.0579***	0.0631**
	(0.0137)	(0.0203)
Emotional stability (std.dev.)	0.0888***	0.101***
	(0.0126)	(0.0192)
Openness to experience (std.dev.)	0.122***	0.135***
	(0.0142)	(0.0202)
Log income (mean)	-0.231***	-0.245***
	(0.0110)	(0.0156)
Log income (std.dev.)	0.0804***	0.0927***
	(0.00777)	(0.0106)
Age (mean indiv.)	0.00129***	0.000179
	(0.000265)	(0.000382)
Controlled for waves responded	Yes	No
Observations	11328	5854
Adjusted R^2	0.346	0.358

Table S2: Robustness of our models. Comparison between results from main (unbalanced) sample and the strongly balanced sample.
